# Perovskite Piezoelectric-Based Flexible Energy Harvesters for Self-Powered Implantable and Wearable IoT Devices

**DOI:** 10.3390/s22239506

**Published:** 2022-12-05

**Authors:** Srinivas Pattipaka, Young Min Bae, Chang Kyu Jeong, Kwi-Il Park, Geon-Tae Hwang

**Affiliations:** 1 Department of Materials Science and Engineering, Pukyong National University, 45, Yongso-ro, Nam-Gu, Busan 48513, Republic of Korea; 2 Division of Advanced Materials Engineering, Jeonbuk National University, Jeonju 54896, Republic of Korea; 3 School of Materials Science and Engineering, Kyungpook National University, Daegu 41566, Republic of Korea

**Keywords:** piezoelectric conversion, energy harvesting, nanogenerator, perovskite piezoelectric composite and thin film, lifetime extension, wireless sensor nodes, self-powered devices

## Abstract

In the ongoing fourth industrial revolution, the internet of things (IoT) will play a crucial role in collecting and analyzing information related to human healthcare, public safety, environmental monitoring and home/industrial automation. Even though conventional batteries are widely used to operate IoT devices as a power source, these batteries have a drawback of limited capacity, which impedes broad commercialization of the IoT. In this regard, piezoelectric energy harvesting technology has attracted a great deal of attention because piezoelectric materials can convert electricity from mechanical and vibrational movements in the ambient environment. In particular, piezoelectric-based flexible energy harvesters can precisely harvest tiny mechanical movements of muscles and internal organs from the human body to produce electricity. These inherent properties of flexible piezoelectric harvesters make it possible to eliminate conventional batteries for lifetime extension of implantable and wearable IoTs. This paper describes the progress of piezoelectric perovskite material-based flexible energy harvesters for self-powered IoT devices for biomedical/wearable electronics over the last decade.

## 1. Introduction

In the ongoing era of the fourth industrial revolution, internet of things (IoT) devices (wireless sensors, a signal processor, a power management circuit and a data logger) are a key technology in collecting and controlling all the information related to biomedical human healthcare (heart and pulse rate), environmental concerns (temperature and humidity), industrial manufacturing, public safety (health condition of mine workers), risk identification (detection of toxic gases and fire, built-in GPS tracker provides the current location of miners) and managing greenhouses in smart cities [1,2,3,4]. To operate IoT devices, batteries are widely utilized as the electric power supply and should be repeatably replaced owing to their limited energy capacity [5]. Nevertheless, the battery maintenance of billions of future IoT devices may not be realistic due to the human effort and expense required, which is the main issue for practical demonstration of IoT sensors [6]. In particular, implantable biomedical IoT devices for healthcare monitoring and disease diagnosis require repeated surgeries for replacement of batteries every several years, which could impose physical, psychological and financial burdens onto patients [7].

Energy harvesters have been attracting much attention over the past decade that can convert ambient energy in the environment, such as mechanical vibrations, heat, fluid flows, electromagnetic radiation, radio waves and in vivo energies into electrical energy for low-power applications [8]. Energy harvesting is a sustainable power source which can be utilized in wireless sensor networks (WSNs), mobile electronics and implantable and wearable IoT devices, eliminating the need for network-based energy, conventional batteries, cables and minimizing maintenance and ecological costs [9]. In energy harvesting, various energy conversion mechanisms have been investigated such as piezoelectric [10,11], pyroelectric [12], photovoltaic [13], triboelectric [14] and magneto-mechano-electric [15]. Of these, piezoelectric energy harvesters have received great attention in the scientific community since piezoelectric materials can precisely convert tiny mechanical/biomechanical movements such as muscle contraction/relaxation and cardiac/lung movements into electricity due to their high electromechanical coupling factor and piezoelectric coefficient [8,16,17,18]. The important application of piezoelectric energy harvesters is in wireless sensor networks. As the number of sensor nodes deployed in various fields has increased continuously, as well as a decrease in node size and power requirement, this makes it possible to harvest ambient energy for sustainable power supply to sensor nodes. For example, wireless communication of sensors driven by piezoelectric energy harvesters can be used to monitor car tire pressure [19].

Moreover, piezoelectric harvesters can utilize in vivo energies such as blood flow, heartbeat and muscle stretching for biomedical devices, such as cardiac pacemakers and brain stimulators for diagnosing heart rate [20]. In addition to in vivo energies, piezoelectric energy harvesters can be installed on footwear, knees, elbows, wrists and fingers to harness biomechanical energy produced by physical activities such as footfalls, hand swings, finger tapping, etc. This energy can be used to power a wide range of electronic devices, including LED lights, wristwatches, mobile phones and implantable biomedical devices [21]. Ambient mechanical energy is the most ubiquitous form of energy and can be utilized to self-power WSNs through piezoelectric effect [8,22]. However, rigid and bulky harvesters have limited utility as implantable or wearable energy sources inside or outside the human body owing to incongruent contact with the curved and corrugated surface of the human skin, muscles and organs. Piezoelectric-based flexible material, lightweight and slim energy harvesting devices on thin plastic films, have been widely developed over the last decade to achieve conformal settlement and energy scavenging on human muscle and organ surfaces to resolve the aforementioned issues [23]. In particular, various research groups around the world have studied high-performance flexible piezoelectric energy harvesters by utilizing piezoelectric materials, which can generate electric energy by tiny mechanical deformations and irregular vibrations, thus potentially enabling various implantable and wearable self-powered IoT devices [24]. The utilization of flexible piezoelectric devices is not only limited to energy harvesting applications but also include mechanical self-powered sensors, which could be utilized to demonstrate future in-vivo biomedical sensors for real-time monitoring of the body condition.

Since 2010, new film-type flexible piezoelectric energy harvesters have been fabricated by using perovskite-structured inorganic BaTiO_3_ (BT), Pb(Zr,Ti)O_3_ (PZT), Pb(Mg_1/3_Nb_2/3_)O_3_–PbTiO_3_ (PMN-PT) and Pb(Mg_1/3_Nb_2/3_)O_3_–Pb(Zr,Ti)O_3_ (PMN-PZT) films with highly piezoelectric constants for outstanding energy conversion efficiency [25,26,27,28]. The inorganic-based piezoelectric films are transferred onto flexible plastic films by various transfer processes after high temperature annealing for crystallization. The perovskite piezoelectric film flexible harvesters show the best output performance among flexible piezoelectric materials. On the other hand, composite type flexible piezoelectric energy harvesters with large area, low cost and stable mechanical properties have also been widely studied [29,30]. These piezoelectric composites composed of nanoscale (or microscale) piezoelectric materials and polymer matrix are fabricated by simply casting a piezoelectric composite onto flexible plastic films. Various research teams have fabricated composite type piezoelectric energy harvesters by using high-performance or lead-free inorganic-based perovskite piezoelectric materials to demonstrate flexible composite harvesters, as well as ultra-stretchable elastic composite generators. This review paper presents a brief overview of flexible piezoelectric composite and film-based energy harvesters reported over the last decade to realize self-powered implantable and wearable IoT electronics. In particular, this manuscript is focused on introducing perovskite-based flexible piezoelectric materials for energy conversion applications, including self-powered energy sources, biomedical stimulators and mechanical sensors. This technology could offer novel solutions to overcome the challenging issues of flexible piezoelectric harvesters.

## 2. Flexible Piezoelectric Composite and Film-Based Energy Harvesters for Self-Powered IoTs and Wearable/Biomedical Electronic Devices

### 2.1. Flexible Piezoelectric Composite-Based Energy Harvesters

#### 2.1.1. Role of Graphitic Carbons with Perovskite Piezoelectric Nanoparticles for Flexible and High-Performance Lead-Free Nanocomposite Generators

**Figure 1:**
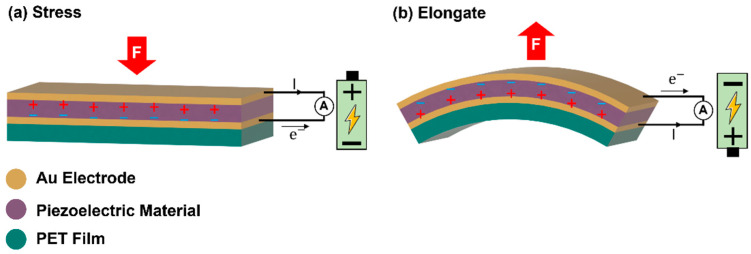
Schematic illustration for the operation mechanism of piezoelectric energy harvester. Generation of electricity by (**a**) applying stress and (**b**) releasing stress.

Energy harvesting technology has received considerable attention because of its simple structure and the ease of application of piezoelectric energy harvesters, which are composed of nano- or micro-structured piezoelectric materials/composites and films [17,31,32,33,34]. When a piezoelectric energy harvester responds to an external stress/pressure/bending/stretching, the piezoelectric material is strained, which results in electric potential and charge through a direct piezoelectric effect, as schematically shown in Figure 1 [17,25]. Wang and co-workers [17,33,35,36,37,38] developed flexible piezoelectric energy harvester (called nanogenerator) devices using piezoelectric ZnO nanowire arrays for application to light emitting diodes [37], liquid crystal displays [38] and wireless data transmission [33]. These nanogenerators can generate electricity from tiny mechanical movements of muscles, heartbeat, blood flow and internal organs from live rats [39,40].

**Figure 2:**
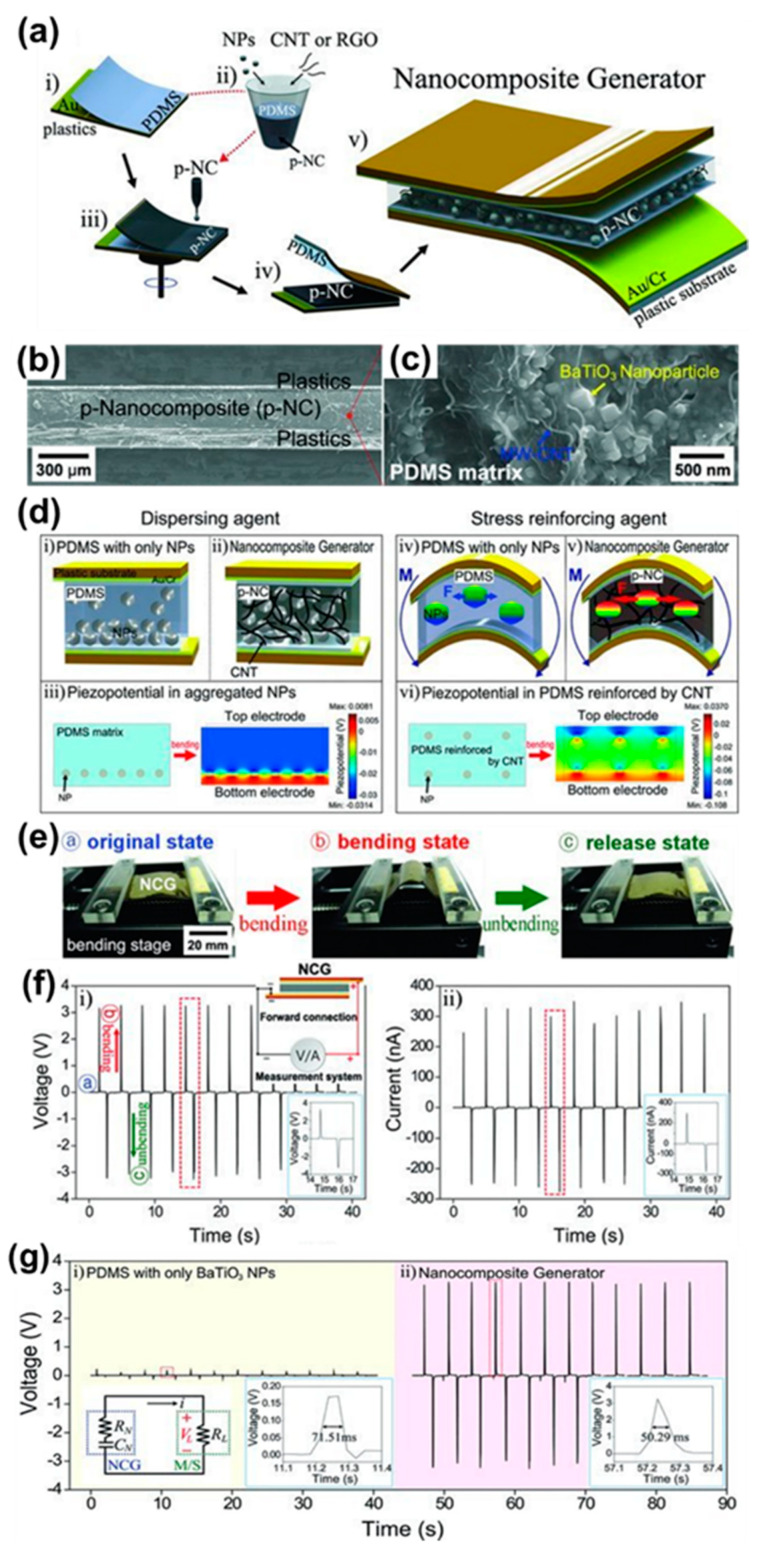
(**a**) Schematic diagram of the fabrication process for NCG device. (**b**) Cross−sectional SEM image of NCG device. (**c**) Magnified cross−sectional SEM image of the piezoelectric nanocomposite. (**d**) Schematic illustration of the cross−sectional structure of NCG device and estimated piezoelectric distributions for investigating the role of CNTs as (**i**–**iii**) a dispersing agent and (**iv**–**vi**) a stress reinforcing agent. (**e**) Photographs of NCG device in original, bending and release states. (**f**) Voltage and current response of NCG device in forward connection. (**g**) Voltage response of a device containing only BT NPs and a NCG device. The bottom-left inset of indicates the equivalent circuit diagram of NCG device. The bottom-right insets indicate the magnified voltage response by the mechanical bending motion. Reproduced with permission [29].

There have been many attempts to prepare piezoelectric film-based harvesters using perovskite structure piezoelectric materials (PZT and BT) due to their outstanding piezoelectric responses [25,41]. Park et al. [25] demonstrated a lead-free BT thin film nanogenerator by employing a high-temperature annealing transfer method from bulk substrates onto flexible substrates and this generated higher power density than other devices with the same structure [35]. In 2012 [29], a research group developed a flexible nanocomposite generator (NCG) device using perovskite piezoelectric BT nanoparticles (NPs) and graphitic carbon for self-powered energy systems. The fabrication process of the NCG is schematically shown in Figure 2a. The BT NPs (12 wt%) were synthesized by a hydrothermal reaction method and then mixed with graphitic carbons (1 wt%) such as single and multi-walled carbon nanotube (SW and MW-CNTs) and reduced graphene oxide (RGO) in a polydimethylsiloxane (PDMS) solution by mechanical agitation to form a piezoelectric nanocomposite (p-NC). The well-dispersed p-NC was coated onto an Au-coated plastic substrate by a spin-casting technique and then dried in an oven. The top and bottom electrodes were deposited on flexible substrates by radio frequency magnetron sputtering technique. Cu wires were attached to these electrodes by Ag paste for the characterization of the NCG device.

Figure 2b shows a cross-sectional SEM image of p-NC with a thickness of 250 μm sandwiched between top and bottom Au/Cr coated plastic substrates. SEM studies revealed that BT NPs and MW-CNTs are rectangular (grain size of 100 nm) and cylindrical in shape (diameter of 5 to 20 nm and length of 10 μm) with a uniform microstructure and are well distributed in the PDMS matrix, as shown in Figure 2c. A finite element analysis was performed using COMSOL simulation software to calculate the piezoelectric distributions inside the NCG device by considering a rectangular model that is composed of six BT NPs in the PDMS matrix. The piezo-potential for the tensile strain was found to be 0.33% on the *X*-axis and is generated by BT NPs across the top and bottom sides of the PDMS matrix. Further, the NCG device was characterized with and without CNTs to exploit its key role in the NCG. In NCG, the role of BT NPs is to generate a piezoelectric potential by applying mechanical stress/bending, which acts as an energy generation source, whereas the CNTs act as dispersant and stress reinforcing agents. It has been very challenging to disperse BT NPs uniformly in the PDMS matrix for high performance of a NCG. Aggregation is prevented only by BT NPs and such aggregation results in poor dispersion (Figure 2(d-i)) and low output voltage. The BT NPs were well dispersed (Figure 2(d-ii,c)) with CNTs by forming a complex mixture, which leads to high output voltage. Figure 2(d-iii,d-vi) show the piezoelectric distribution of aggregated NPs and PDMS reinforced by CNTs. Another role of CNTs is to reinforce the stress applied to NPs by increasing the composite stress. The increment is a result of a change in the mechanical responses of the composite material [42,43]. The small NPs can be significantly stressed because the CNTs and BT NPs in the PDMS matrix are well mixed and entangled, as schematically shown in Figure 2(d-iv,d-v).

The generated output voltage and current response of the NCG during periodic bending and unbending motions in the forward connection is shown in Figure 2f. The NCG generated an open circuit voltage (V_oc_) of 3.2 V and a short circuit current (I_sc_) of 350 nA during bending and unbending cycles. The generated voltage and currents are much higher compared to the BT thin film nanogenerator (V_oc_ = 1 V) and I_sc_ = 26 nA) reported by Park et al. [25]. In addition, the conduction paths created by the CNT networks can lower the internal resistance of the device [44], resulting in a short voltage lifetime and high output characteristics [45,46]. The NCG device was generated a sharper peak of output voltage than the device without CNTs, as shown in the bottom-right insets of Figure 2(g-i,ii). The equivalent circuit diagram of the device is displayed in the bottom-left inset of Figure 2(g-i), where *R_N_* is the internal resistance, *C_N_* is the capacitance and *R_L_* is the load resistance of the system, respectively. The piezoelectric charges are generated at two ends of the device by mechanical deformation and then removed during the RC discharging process with a time constant (*τ*) given by the following Equation (1) [45,46].

(1) $$\tau =\left(R_{N}+R_{L}\right)\;\cdot \;C_{N}$$

The output voltage lifetime, defined as the full width at half maximum (FWHM) of the output voltage, is 50.29 ms and 71.51 ms for NCG and NCG without CNTs, respectively. The NCG with CNTs shows a shorter output voltage lifetime than NCG without CNTs. This confirms that a short lifetime of the NCG caused by CNT additions results in an increase in output voltage. The experimental characterization is well supported by the theoretically analyzed results showing the piezo-potential, principle of power generation and the role of CNTs in a NCG device. This technique successfully overcomes the size-related limitations of previous nanogenerators, enabling simple, low-cost, large-scale self-powered energy systems. These findings significantly enhance the viability of self-powered energy systems for application in consumer electronics, sensor networks and indoor energy harvesting.

#### 2.1.2. Role of Metal Nanorods with Piezoelectric Particles for Flexible and High-Performance Lead-Free Nanocomposite Generator

Energy harvesting from mechanical energy sources such as human walking, transportation movement and sound waves is a potential candidate for energy generation with better accessibility and eco-compatibility [22,35,47]. The first piezoelectric nanogenerators were developed by directly converting energy from mechanical motion to electrical power in the order of milliwatts (mWs) for applications in wireless communication sensor networks and self-powered flexible electronic devices such as biological and environmental monitoring systems [17,22,48]. The previous section introduced a flexible NCG made with perovskite structured piezoelectric BT NPs and CNTs to develop simple and low-cost energy harvesters for self-powered systems [29]. However, it has a few drawbacks in obtaining high output power, such as the low piezoelectric response of BT NPs and aggregation of CNTs. The (K,Na)NbO_3_ (KNN) is the most attractive perovskite lead-free piezoelectric materials due to its superior piezoelectric, electromechanical responses, biocompatibility and high Curie temperature [49,50,51]. The KNN materials modified by doping elements greatly improved their piezoelectric properties (d_ij_ > 300 pC/N) [50,52], as compared to BT [25], NaNbO_3_ [53], KNbO_3_, [54] and pure KNN (<100 pC/N) [55,56]. Especially, the 0.942 (K_0.480_Na_0.535_)NbO_3_-0.058LiNbO_3_ (KNLN) composition has shown outstanding piezoelectric response (d_33_ = 310 pC/N) [57,58]. In order to overcome these drawbacks, Jeong et al. [59] demonstrated a high-performance lead-free NCG device using piezoelectric KNLN particles and well-dispersed copper nanorods (Cu NRs). The KNLN particles provide power generation and Cu NRs act as energy enhancers embedded in the PDMS matrix to form p-NC. In p-NC, the key role of Cu NRs is to act as dispersing, reinforcing and conducting agents to achieve high-performance NCG devices.

**Figure 3:**
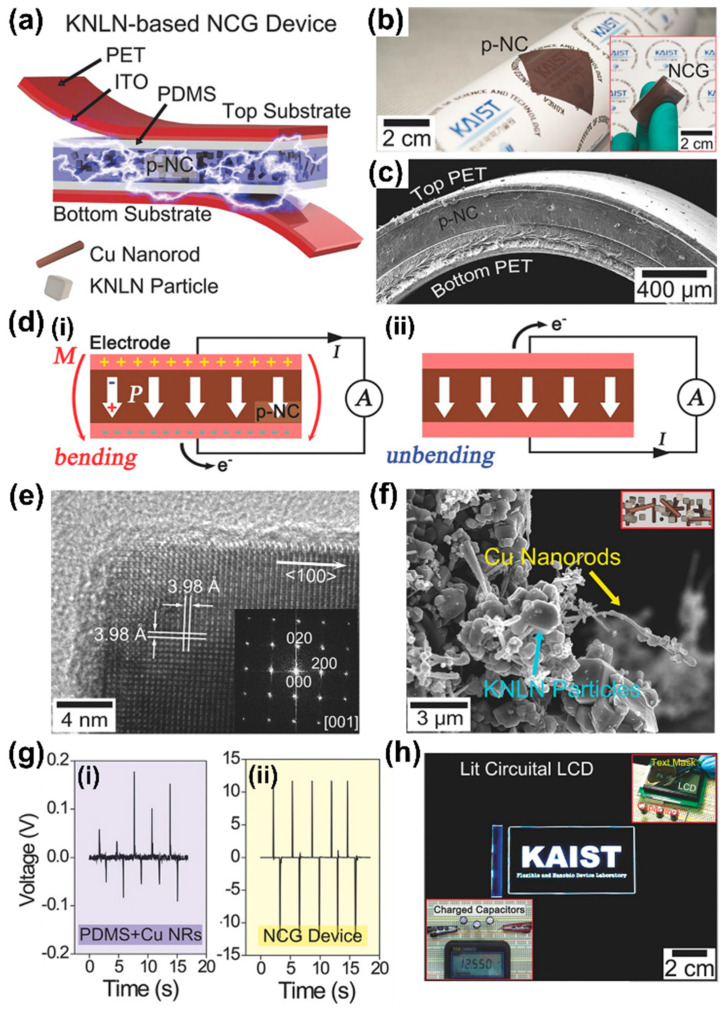
(**a**) Schematic diagram of NCG device containing KNLN particles and Cu NRs. (**b**) Photograph of flexible p−NC layer attached to the curved surface of the paper. (**c**) Cross−sectional SEM image of bent NCG device embedded with KNLN particles. (**d**) Schematic diagram of the power generation mechanism of NCG during (**i**) bending and (**ii**) unbending state. (**e**) HRTEM image of FFT analysis (inset Figure) of KNLN particles. (**f**) SEM image of mixed nanomaterials made by KNLN particles and Cu NRs. (**g**) Generated voltage from devices made of (**i**) PDMS and Cu NRs and (**ii**) NCG devices composed of Cu NRs and KNLN particles. (**h**) Photograph of commercial LCD device operated by charged capacitors by NCG (bottom inset) and LCD device circuit with a text mask (top inset). Reproduced with permission [59].

A schematic illustration of the NCG device structure with KNLN particles and Cu NRs forming a well-distributed p-NC layer in the device is shown in Figure 3a. The indium tin oxide (ITO) coated electrodes on a polyethylene terephthalate (PET) flexible substrate act as a current collector, whereas the PDMS layer is used to avoid electrical breakdown during the high voltage poling process and extreme mechanical deformation. Figure 3b shows a photograph of flexible p-NC attached to a rolled paper, which is placed between the top and bottom ITO-coated PET substrates for a bendable device (inset of Figure 3b). A cross-sectional SEM image of a bent device prepared by p-NC with a thickness of 250 μm is shown in Figure 3c. The power generation mechanism of the device is schematically depicted in Figure 3d. When the device is subjected to external bending, current flow is generated between two electrodes owing to the positive piezo potential produced on the top side (Figure 3(d-i)). During the unbending of the device, the accumulated charges are returned to the original state because of the disappearance of the piezo-potential, which causes output electric signals in the reverse direction (Figure 3(d-ii)).

Figure 3e shows the crystal structure of KNLN particles examined by high-resolution transmission electron microscopy (HRTEM), where a fast-Fourier transform (FFT) analysis revealed a single crystal structure (inset of Figure 3e). The interplanar separation (3.98 Å) shown in the HRTEM image corresponds to the (010) and (100) crystallographic planes and has crystallographic lattice constants of a = b in the tetragonal crystal symmetry of KNLN particles [60]. A SEM image of KNLN particles and Cu NRs is shown in Figure 3f. These studies revealed that well-dispersed KNLN particles and Cu NRs have a length of 5 µm and diameter of 200 to 400 nm. The generated voltage responses from NCG devices fabricated using a PDMS-Cu NRs composite and NCG device are shown in Figure 3g. There is a weak electrical signal from the device containing Cu NRs embedded in the PDMS layer without KNLN particles due to several electrostatic charges at electrodes (Figure 3(g-i)). The output voltage response is lower than that of the NCG device with both Cu-NRs and KNLN particles (Figure 3(g-ii)), which is attributed to the absence of dispersing and reinforcing agents. Additionally, the Cu NRs in the p-NC can act as nano-electrical bridges, resulting in short voltage lifetimes for better output performance [29,61]. The generated voltages of 12.55 V from the NCG device are stored in capacitors and successfully operated a commercial LCD device incorporated with a metal-patterned text mask (Figure 3h). These results demonstrated a flexible, high-performance and lead-free NCG device incorporating piezoelectric KNLN particles and well-dispersed CuNRs that generate higher power than previously reported, flexible and lead-free composite-based piezoelectric harvesters [62,63]. The lead-free NCG technology and higher power are significant developments in the research on self-powered flexible energy sources and can enable bio-eco-compatible flexible electronics and sensor networks for ubiquitous wireless communication.

#### 2.1.3. Role of Metal Nanowire Electrodes on Piezoelectric Particles and Carbon Nanotubes for Hyper-Stretchable and Elastic-Composite Generator

Stretchable electronics that exhibit elastic properties in response to large strain deformation have attracted attention for use in a variety of new applications, including biomimetic lenses, artificial electronic skins, biomedical devices, artificial electronic skins and body sensor networks [64,65,66]. Even though various approaches have been attempted to fabricate viable stretchable or wearable electronics and batteries, a challenging task is in power supplies, which require similar elastic responses in order to be co-integrate with stretchable devices. Recently, high-performance flexible piezoelectric energy harvesters reported using perovskite piezoelectric films include BT [25], PZT [24] and PbMg_1/3_Nb_2/3_O_3_-PbTiO_3_ (PMN-PT) [67] for self-powered flexible electronics and mW-scale energy harvesting. Despite that stretchable NCGs had been conceptualized as stretchy piezoelectric devices, a fully functional stretchable NCG had not been realized due to the lack of suitable flexible electrodes and a sturdy composite matrix. In this regard, Jeong et al. [68] investigated a simple and uncomplicated route to develop a high-performance, hyper-stretchable and elastic-composite generator (SEG) using very long Ag nanowires (VAgNWs) stretchable electrodes.

**Figure 4:**
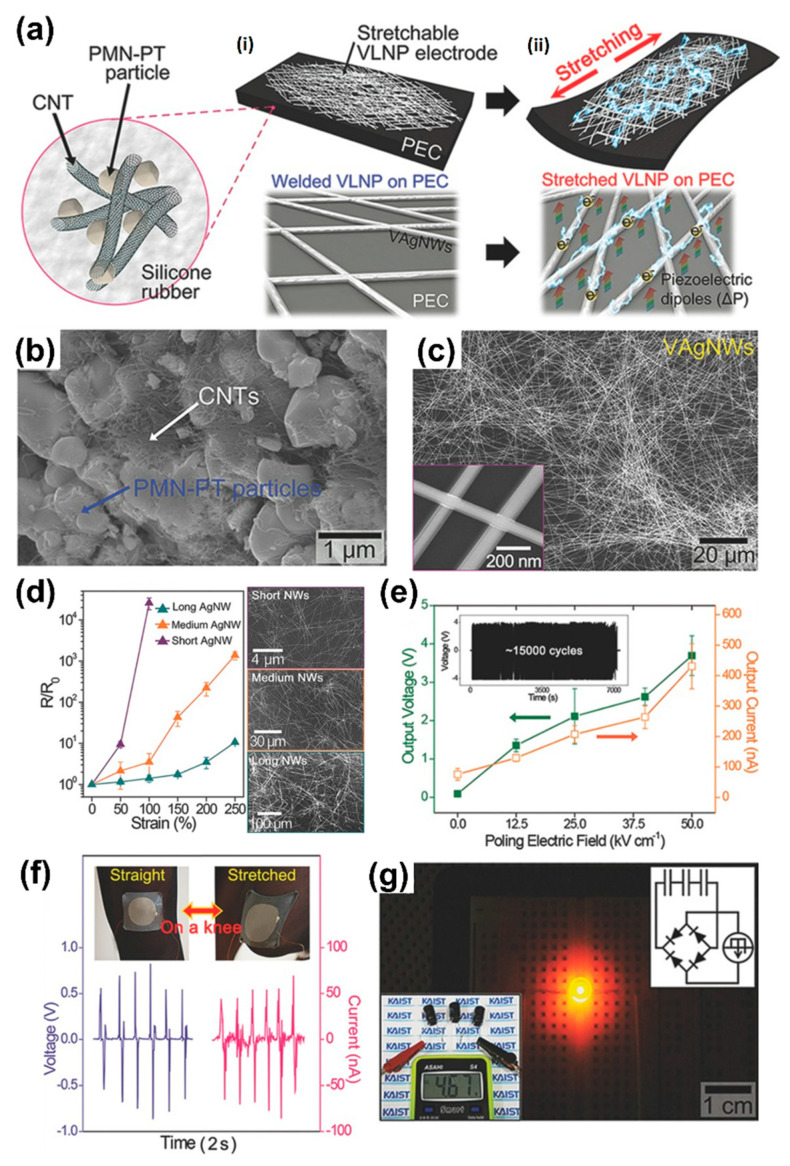
(**a**) Schematic diagram of the hyper-stretchable and deformable NCG. (**a**-**i**) VAgNWs transferred onto both sides of PEC made of PMN−PT particles, MW−CNTs and Si−rubber matrix for the fabrication of stretchable electrodes. (**a**-**ii**) Generation of electricity of SEG when it is stretching and deforming. The bottom figures show magnified schematics of welded VLNP and stretched VLNP on PEC. (**b**) SEM image of well−dispersed PMN−PT particles and MW−CNTs. (**c**) Overall and magnified (inset) SEM images of VAgNWs synthesized by SMG method. (**d**) The change of relative resistance with strain for short AgNW, medium AgNW and long AgNW percolation network electrodes, calculated by a COMOSOL simulation. (**e**) Generated output voltage and current versus polling electric field of the stretched SEG device. The durability test of SEG for 15,000 cycles (inset). (**f**) The voltage and current are generated from SEG on the stocking by bending and straightening the knee. (**g**) Photograph shows a commercial LED bulb turned on by the stored energy in capacitors (bottom inset), charged by generated energy from SEG. Reproduced with permission [68].

A schematic diagram of the SEG structure made of a hyper-stretchable piezoelectric elastic composite (PEC) and very long nanowire percolation (VLNP) electrodes is shown in Figure 4a. Ecoflex silicone rubber used for the matrix of PECs, referred to as an ultra-stretchable elastomer (~900% in elastic deformation), is better than a PDMS matrix (<100% in plastic deformation). The optimized PEC of PMN-PT particles and MW-CNTs were mixed and dispersed in silicon rubber for better molding. The successive multistep growth method (SMG) is employed to synthesize VAgNWs, which were filtered by a suction pressure process and transferred onto both sides of PEC (Figure 4(a-i)). The VAgNWs are capable of creating a highly percolated network nanostructure that functions as a stretchable electrode with good conductivity and stability [69]. Further, nano-welding was performed between VAgNWs to obtain low resistance and VLNP electrodes were welded onto PEC, as shown in the bottom of magnified Figure 4a-i. When PEC is stretched by applying force, the piezoelectric particles in the matrix can be stressed by the mechanical strain, which results in a variation of piezoelectric dipoles (ΔP) in the PEC. The change of dipole moments leads the VLNP electrodes to accumulate/push electrons via an external load, producing output electricity, as shown at the bottom of Figure 4(a-ii)). Finally, a hyper- stretchable SEG was fabricated to generate electricity (Figure 4(a-ii)). SEM studies revealed well-dispersed PMN-PT particles and MW-CNTs in the PEC (Figure 4b). The CNTs act as dispersing, reinforcing and bridging agents in the p-NC, as discussed in the above section. Appropriate length of AgNWs is essential for the conductive NW percolation network to attain good electrical conductivity and mechanical stability [69]. The chemically synthesized metallic NWs are limited to 20 µm in length, showing weak electrical and mechanical responses [70]. Hence, a novel SMG synthesis method was developed to enhance the length of AgNWs to a maximum value of 500 µm (Figure 4c).

A COMSOL simulation analysis was performed to understand the structural mechanics of deformed NW networks related to the length of AgNWs. The relative resistance (R/R_0_) of AgNW was changed for various lengths: a short AgNW electrode (average length of 3 µm), medium AgNW electrode (average length of 20 µm) and very long AgNW percolated electrode, as shown in Figure 4d. Under large strain, very long AgNW percolated electrodes on PEC displayed highly stable electrical and mechanical responses compared to short and medium AgNW electrodes. The short AgNW network electrode displayed a sharp increase in resistance because of fracture induced by deformation. The medium AgNW network electrode has a better response than the short NW network electrode, but does not provide better stretchability. These results confirmed the importance of VAgNWs for ultra-stretchable electronic and energy devices, including the realization of stable reversibility and outstanding elasticity.

The generated voltage and current from the SEG device at a poling electric field from 0 to 50 kV/cm is shown in Figure 4e and was enhanced to 4 V and 500 nA at 50 kV/cm. The SEG’s output voltage was consistently measured without degradation during approximately 15,000 repeated stretching motions. This indicates that the stretchable VAgNW electrodes-based SEG displayed better electrical and reversible responses under reciprocating 200% deformation. The SEG was stitched onto the knee of a nylon stocking to emphasize the stretchability and viability of this stretchy energy harvester for wearable systems. The generated voltage was 0.7 V and current was 50 nA from the SEG during biomechanical stretching motions of kneeling and releasing movement, as shown in Figure 4f. In addition, the generated output power from SEG was used to operate a commercial LED bulb, which was lighted by charged capacitors (bottom inset), as shown in Figure 4g. These results demonstrated a hyper-stretchable SEG composed of VLNP network electrodes and PEC. The SEG comprised of well-dispersed PMN-PT particles and MW-CNTS with a Si-rubber matrix generated higher voltage and a current of 4 V and 500 nA with stretch ability of 200%. This robust energy conversion and hyper-stretchable SEG provides a novel platform for microelectromechanical systems (MEMS) [71], triboelectric applications [72], wearable devices [73] and self-powered stretchable electronics.

#### 2.1.4. Bioinspired Piezoelectric Composite Generator for High-Performance Energy Harvesting

The piezoelectric composite generators (PCGs) have been fabricated by dispersion of nano/microparticles, metal nanowires/nanorods/fillers with polymer matrix, and have been identified as good candidates for the applications in energy harvesting devices due to their advantages of inorganic particles/fillers with polymer matrix including high piezoelectricity, mechanical stability and ease of processing [30,74,75]. However, they have a few drawbacks, such as inhomogeneous filler dispersion and ineffective stress transfer from external forces to internal piezoceramic components, which restrict the performance of the energy harvester. In recent years, novel hierarchical nanocomposites with improved mechanical properties have been investigated by inspiring the structures of biomaterials like bones, silks, shells, etc. [76,77,78,79]. The sea sponge is a simple multi-cellular living organism and is a highly attractive composite structure, which is composed of soft fibrils and hard skeletons with three-dimensional (3D) porous configurations to show high elasticity and strong toughness [80]. To overcome such drawbacks, Zhang et al. [81] fabricated PCGs using sea sponge and 0.5 (Ba_0.7_Ca_0.3_)TiO_3_–0.5 Ba(Zr_0.2_Ti_0.8_)O_3_ (BCZT) piezoceramics to enhance the mechanical and piezoelectric performance of the energy harvester. The BCZT is a well-known lead-free piezoelectric material, which is a good candidate for wearable and bio-related applications due to its outstanding piezoelectric responses [74,82].

**Figure 5:**
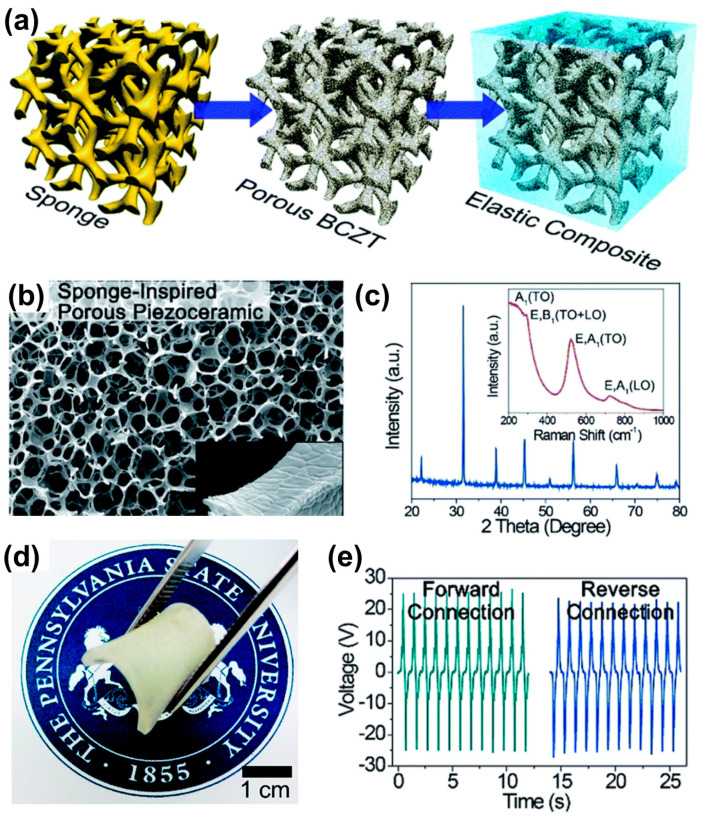
(**a**) Schematic diagram of the synthesis process of the sea sponge−inspired 3D piezoelectric composite. (**b**) SEM micrograph of the BCZT porous structure. A cross−sectional SEM micrograph of the porous BCZT. (**c**) XRD pattern and Raman spectrum (inset) of the sea sponge−inspired BCZT porous structure. (**d**) A photograph of elastic PCG. (**e**) Generated open−circuit voltage signals from PCG by compressing and releasing under forward and reverse connections. Reproduced with permission [81].

The bioinspired piezoelectric composite structure was synthesized using a sponge-template approach, as schematically shown in Figure 5a. First, the sol-gel synthesis process is employed to prepare 0.5 (Ba_0.7_Ca_0.3_)TiO_3_–0.5 Ba(Zr_0.2_Ti_0.8_)O_3_ piezoelectric composite solution. The polyurethane (PU) sponge foam was immersed entirely in the BCZT solution for 5 min. Further, it was partially dried in an oven at 60 °C to remove excess solvents and then by calcination at 1200 °C. The sponge acts as a sacrifice template skeleton during the calcination and burns completely, while the BCZT sol-gel is pyrolyzed and crystallizes in the shape of the 3D porous structure of the sponge. The PDMS elastomer was infiltrated into the 3D interconnected porous BCZT (54 wt%) to prepare the 3D BCZT-PDMS composites. The fabrication process of this composite generator is similar to the earlier reports [29,30,74,75,83,84].

The SEM image of the bioinspired porous BCZT composite structure displayed a uniform pore size (100–300 µm) of the 3D interconnected ceramics, which is similar to natural sea sponges. The average grain size of BCZT was found to be 0.5–300 µm, as shown in inset Figure 5b. It is evident that the template method produces the piezoelectric structure like a sea sponge while also removing the template and building the ceramic scaffold without undesired collapses and cracks. The XRD pattern of bioinspired 3D BCZT composite confirms a pure perovskite phase of BCZT without any secondary phase, as shown in Figure 5c. The Raman active modes appeared at 300 and 700 cm^−1^ are associated with the E, B1 (TO + LO) modes and E, A1 (LO) modes correspond to the tetragonal perovskite phase of BaTiO_3_ (inset of Figure 5c) [74,85]. The Raman bands are broadened, which reveals the tetragonal phase coexisting with rhombohedral phase. As compared to the Raman spectra of BaTiO_3_, the intensities of Raman modes of BCZT are broadened, which confirms the coexistence of a morphotropic phase boundary between the tetragonal and rhombohedral phase and is consistent with previously reported Raman spectra of BCZT [86]. The perovskite piezoelectric materials with MPB compositions have been well recognized for enabling strengthened piezoelectricity [84]. A photograph of a synthesized BCZT composite with infiltrated PDMS matrix is shown in Figure 5d, exhibiting high elasticity and bendability even at a bulk-size thickness of 1 mm. The generated V_oc_ of 25 V (I_sc_ = 550 nA/cm^2^ and P = 2.6 µW/cm^2^) from bioinspired PCG is obtained by compressing and releasing under forward and reverse connection, as shown in Figure 5e. This power density is 16 times more than that of conventional particle-based PCGs [74,83,84,87]. In addition, this PCG generated an output voltage of 5 V and displayed 30 times higher strain-voltage efficiency by stretching than previously reported flexible piezoelectric energy harvesters [30,74,75].

#### 2.1.5. Dislocation-Induced Nanodomains in Piezoelectric Single-Crystal Micro-Cuboids for Transparent and Flexible Lead-Free Piezoelectric Pressure Sensor

The relaxor ferroelectrics (RFEs) have attracted attention due to their outstanding dielectric, ferroelectric, piezoelectric and electromechanical properties, mainly in lead-based perovskites [88,89]. Generally, the electromechanical responses of single-crystal RFEs are superior to those of polycrystalline ceramics. The outstanding properties of RFEs mainly arise from the presence of nanodomains (NDs)/polar nanoregions (PNRs) [90,91,92]. However, the discovered approaches for the formation of NDs/PNRs basically depend on the local composition inhomogeneity [93,94]. These limited approaches might prevent facile nanodomain evolution and wide applications of various RFEs. In this regard, Park et al. [95] reported a novel and simple molten salt (KF) synthetic method for dislocation-induced NDs and K_0.4255_Na_0.5201_Li_0.0394_Ba_0.01_)NbO_3_ (KNN) single -crystal micro-cuboids with RFE properties.

**Figure 6:**
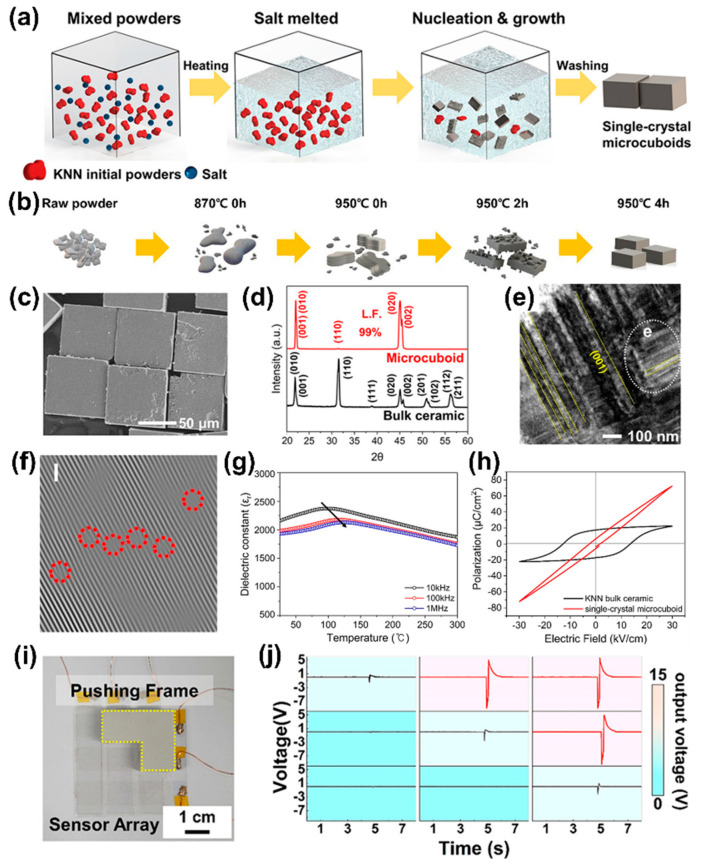
(**a**) Schematics for the synthesis process of KNN single−crystal micro−cuboids by the molten salt method. (**b**) Schematic diagram of the morphological evolution from raw powders to 3D micro−cuboids during the synthesis process. (**c**) SEM image of micro−cuboids. (**d**) X−ray diffraction pattern of micro−cuboids and bulk ceramic sample. (**e**) TEM image of (001) domain wall. (**f**) IFFT images showing dislocations (red dotted circle) along (001) domain wall. (**g**) Temperature variation of dielectric constant (ε_r_) of the KNN micro−cuboids at different frequencies. (**h**) Polarization−electric field (P-E) hysteresis loops of the single−crystal KNN micro−cuboids and KNN bulk ceramic sample measured at 1 kHz and room temperature. (**i**) Photograph of fabricated transparent, flexible KNN pressure sensor. (**j**) Generated output voltage signals from each sensing unit with color code mapping image by applied pressure. Reproduced with permission [95].

The KNN single-crystal micro-cuboids were prepared by the KF method. Calcinated KNN powders and KF salt were mixed and heated above the melting point of salt, which results in nucleation and growth in an ionic liquid medium, as schematically illustrated in Figure 6a. The liquid environment and ionic polarization force of molten salt enabled rapid mass transfer and high reactant dissolution, resulting in a shorter reaction time. The morphological evolution of KKN single-crystal micro-cuboids from calcinated initial powders at various temperatures and duration times is schematically shown in Figure 6b. Irregular large aggregated particles, stacked layers, 2D plates by island-like growth and 3D micro-cuboids were formed when the temperature was increased from 870 to 950 °C, respectively. These results indicate that the KF salt interacts with the growing surface and changes the surface energy to produce intermediate layer structures [96,97].

A SEM image of KNN single-crystal micro-cuboids is shown in Figure 6c. SEM studies revealed that all the elements of KNN were uniformly distributed over micro-cuboids and flat surfaces, sharp corners and edges with an average length of 80 µm. X-ray diffraction (XRD) confirmed the orthorhombic phase in both KNN micro-cuboids and ceramics, where the single crystal micro-cuboids clearly showed a (001) peak with a high Lotgering factor (L.F ~99%), signifying favored growth along the <001> direction. A TEM analysis of KNN single-crystal micro-cuboids revealed a single-crystal nature and dense strip-like nanodomains (lamellar) with sizes of 10 to 50 nm. Many alternating strip-like nanodomains were found in the single-crystal micro-cuboids along the (001) directions (Figure 6e). The formation of nanodomains is related to misfit dislocations induced during oriented attachment growth [98]. Dislocations can reduce the total free energy of single-crystal micro-cuboids by releasing strain by forming domain walls [99]. Many dislocations were observed near the (001) domain wall (red dotted circles in FFT) and where two domains meet each other (Figure 6f). These results strongly support the mechanism of dislocation-induced nanodomain formation.

The temperature-dependent dielectric properties of the KNN single-crystal micro-cuboids displayed a ferroelectric to paraelectric transition temperature (Curie temperature, T_C_) with a diffused transition and shift of T_C_ towards high temperatures, with a frequency indicating the typical signature of RFEs [100], as shown in Figure 6g. The KNN single-crystal micro-cuboids displayed a slim polarization-electric field (P-E) hysteresis loop with a large, maximum polarization (P_max_) of 72 μC/cm^2^, whereas the KNN bulk ceramic sample showed a fat P-E loop with P_max_ of 22 μC/cm^2^ (Figure 6h). A transparent and flexible pressure sensor fabricated using piezoelectric KNN single-crystal micro-cuboids converts mechanical pressure/force into electrical signals (Figure 6i). The generated output voltage signal from each sensing unit was measured and is shown in the color code of the mapping image by applied pressure (Figure 6j). This result provides that the device, prepared using a simple and low-cost method, can be utilized as a self-powered system to monitor external pressure and human activity via real-time detection [101].

### 2.2. Flexible Piezoelectric Film-Based Energy Harvesters

#### 2.2.1. Transferring Piezoelectric Thin Film onto a Plastic Substrate by Laser Lift-Off Process for a Flexible and High-Efficiency Harvester

Flexible and lightweight energy harvesting devices on a thin film can harvest energy from tiny mechanical movements of wind, water flow, heartbeat, respiration movements and diaphragm activities for electricity not only for use in self-powered systems but also in biomedical devices [22,102,103]. Hu et al. [33] developed a thin film nanogenerator using ZnO NWs on a plastic substrate, generating a high output voltage of 10 V and current of 0.6 µA for operating self-powered data transmission. Piezoelectric polymers (PVDF) have been widely used to fabricate flexible and stretchable energy harvesting devices because of their soft nature [34,46]. Recently, our research group and collaborators fabricated piezoelectric nanocomposite-based flexible nanogenerators to achieve scalable, low-cost and high output [29,30,104]. However, these piezoelectric energy harvesters still show insufficient output performance to operate mW-level consumer electrics because of their low piezoelectrical response and low energy conversion rates owing to the thick piezoelectric polymer layers and sandwiched plastic substrates. In this regard, Park and co-workers demonstrated a PZT thin film on a plastic substrate using a laser lift-off (LLO) process to enhance the energy conversion efficiency of energy harvesting devices [26].

**Figure 7:**
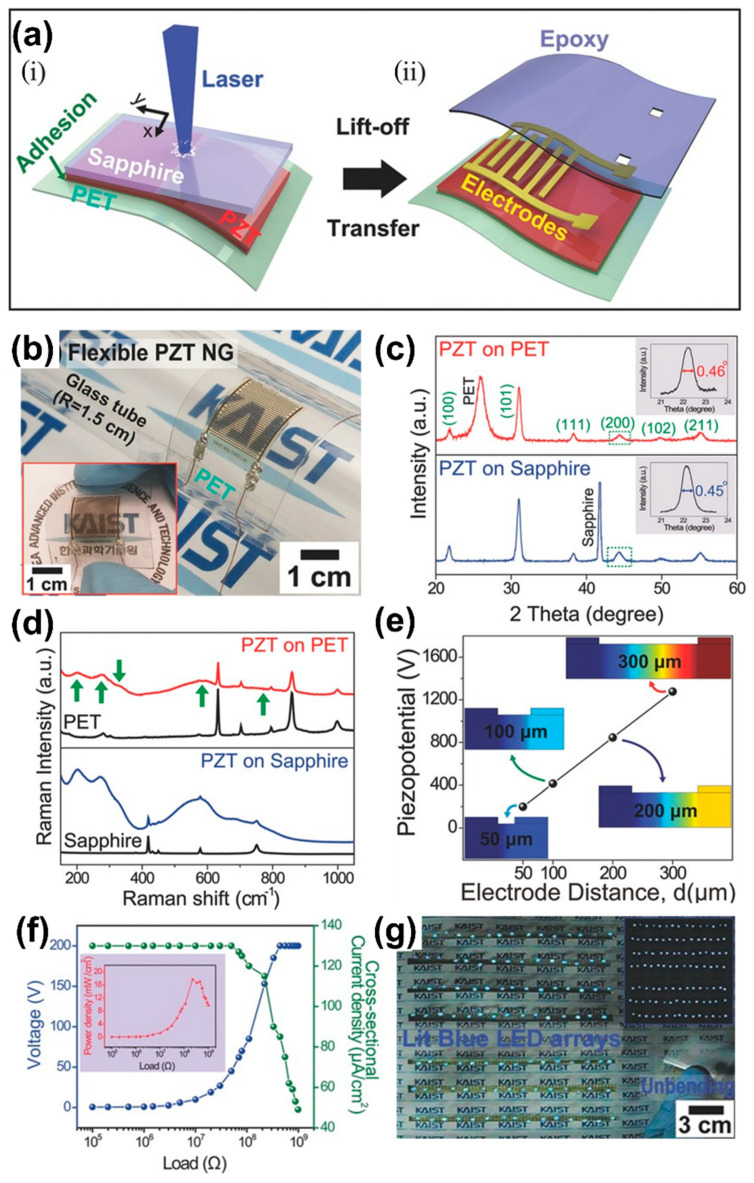
(**a**) Schematic illustration of the fabrication process of PZT thin film harvester by LLO process. (**a**-**i**) PZT thin on transparent sapphire substrate film was transferred onto PET substrate by laser irradiation. (**a**-**ii**) Interdigitated electrodes and a protective layer were coated on PZT thin film for the fabrication of the harvesting device. (**b**) Flexible PZT thin film harvester attached to a glass tube and bent by fingers (inset). (**c**) X-ray diffraction patterns of PZT thin films on sapphire and flexible PET substrate. Magnified view of X-ray diffraction of (200) peak (inset). (**d**) Piezo-potential in PZT film with different electrode distances calculated by the simulation model. (**e**) The calculated piezo-potential in the PZT thin film varied with the distance between adjacent electrodes and increased linearly with an inter-electrode gap on the PZT film. (**f**) The generated voltage and cross-sectional current density of PZT generator as a function of load resistance from 2 kΩ to 1 GΩ. Output power versus load resistance (inset). (**g**) Photograph shows 105 lighted LEDs when the PZT film harvester was unbent by the human finger and driven LEDs in a dark room (inset). Reproduced with permission [26].

The fabrication of a flexible PZT thin film harvester by the LLO process is schematically shown in Figure 7a. The PZT solution was synthesized by a sol-gel method and coated on a sapphire substrate with a thickness of 2 µm by a spin-coater. The PZT coated sapphire substrate was placed on a PET substrate using PU as an adhesive. Ultraviolet (UV) light was then exposed to cure the PU between the PZT film and the PET substrate. An excimer laser with a wavelength of 308 nm was focused on the backside of the sapphire substrate to transfer the PZT film layer from the sapphire substrate. The PZT/PET thin film can be transferred from the sapphire mother substrate (sapphire substrate/PZT/PET) by the irradiation of laser beams, which can pass through the transparent sapphire substrate and locally vaporize the interface between the PZT film and the sapphire substrate, i.e., the LLO process (Figure 7(a-i)). Further, interdigitated electrodes (IDEs) were deposited on a PZT film by a photolithography technique and then SU-8 epoxy was coated to encapsulate the device (Figure 7(a-ii)). The fabricated PZT thin film harvesting device was attached to a glass tube with a radius of 1.5 cm and showed high flexibility and high mechanical stability during bending (Figure 7b).

The XRD patterns of PZT thin films on sapphire and PET substrates before and after the LLO process revealed a polycrystalline perovskite structure and a rocking curve of the (200) peak, as shown in Figure 7c. The Raman modes indicated by green arrows in the Raman spectra confirm the PZT phase (Figure 7d) [105]. Further, piezo-potential distributions of an IDE based PZT thin film device were investigated by a theoretical simulation model. The calculated piezo-potential in the PZT thin film varied with the distance between adjacent electrodes and increased linearly with an inter-electrode gap on the PZT film (Figure 7e). These results supported the fact that the output performance was enhanced by adopting the IDE approach.

Figure 7f shows the generated voltage and cross-sectional current density of a PZT harvester as a function of load resistance. The voltage gradually increased with resistance and then saturated, where the maximum current at low resistance decreased with resistance. The power density estimated by the product of voltage and current was found to be 17.5 mW/cm^2^ at 200 MΩ (inset of Figure 7f). In addition, a large-area PZT thin film energy harvester (3.5 × 3.5 cm) was fabricated, generating a voltage of 200 V and current of 150 µA/cm^2^ during periodical bending and unbending, provided higher output performance than earlier reported piezoelectric harvesters [29,59,68]. Finally, energy by human finger motions was harvested from this PZT device and powered 105 commercial LEDs in series without any external electric source or circuits (Figure 7g). The PZT thin film generator developed by employing the LLO method is promising for self-powered, flexible electronics and biomedical devices for safety, health and environmental monitoring systems.

#### 2.2.2. Transferring Single-Crystalline Piezoelectric Film onto a Plastic Substrate by a Mechanical Exfoliating Process for a Flexible and High-Performance Energy Harvester

For flexible energy harvesters, many researchers have investigated various piezoelectric materials on a plastic substrate, such as ZnO NWs [31,33], BT [25] and PZT thin films [106,107]. Since these harvesters have shown relatively low output current of few µA, their applications are restricted in consumer electronics and biomedical devices. In this regard, a research team demonstrated a flexible and high-performance energy harvester for a self-powered artificial pacemaker using a single crystalline piezoelectric PMN-PT film on a plastic substrate by a mechanical exfoliating process [27].

**Figure 8:**
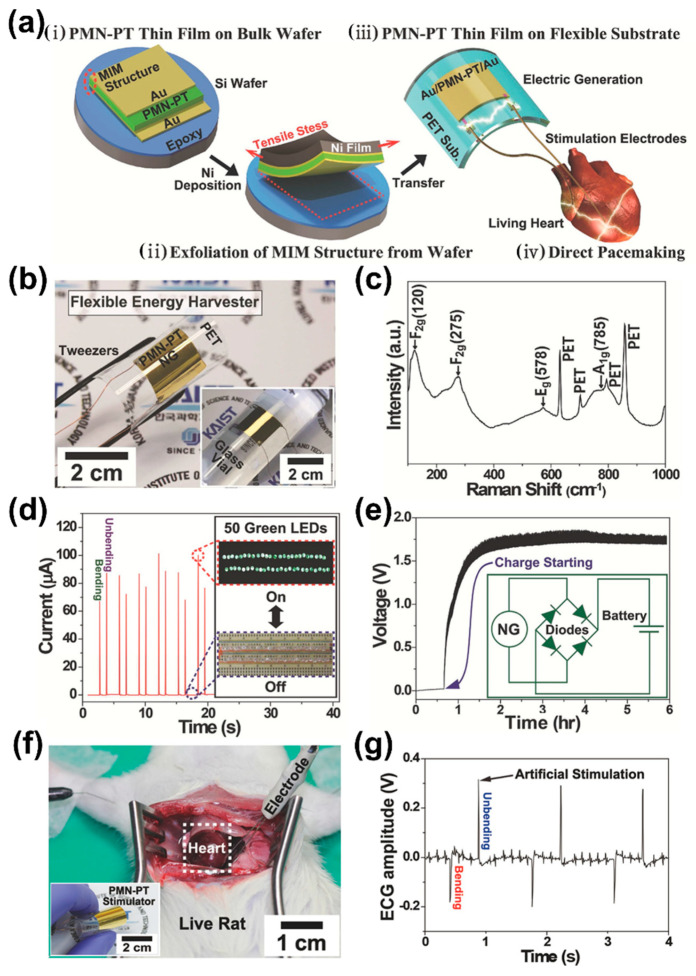
(**a**) Schematic diagram of the fabrication process and stimulating test on a living heart of a flexible PMN−PT film piezoelectric energy harvester. (**b**) Photograph of flexible PMN−PT film NG on PET substrate. The inset figure shows the harvesting device attached on a glass vial. (**c**) Raman vibrational spectrum of PMN−PT film. (**d**) The rectified current of flexible PMN−PT harvester. The inset presents the lighting of 50 green LEDs and corresponding current peaks during the bending and unbending of the harvester. (**e**) Charging response of a coin battery by PMN−PT energy harvester. The inset shows a schematic circuit diagram for energy storage comprising four diodes and a battery. (**f**) Photograph of opened chest of living rat to stimulate its heart. A flexible PMN−PT film stimulator (inset). (**g**) The ECG amplitude of stimuli by bending and unbending of the flexible energy harvester. Reproduced with permission [27].

The fabrication process and stimulation test on the living heart of a flexible PMN-PT film piezoelectric energy harvester were explained in four steps, as schematically shown in Figure 8a. (i) The single crystalline piezoelectric 0.72 PMN–0.28 PT ingot was grown by a modified Bridgman method near the morphotropic phase boundary (MPB) with a rhombohedral crystal structure, which shows high piezoelectric and mechanical coupling factors near the MPB [108,109,110]. The (001)-oriented PMN-PT crystal cut into a square block, Au deposited on PMN-PT plate as a bottom electrode and bonded on a (100) Si wafer using adhesive epoxy. The prepared crystal was poled along the (100) direction at an electric field of 1.8 kV/mm and room temperature for 1 h followed by top electrode deposition. (ii) A mechanical exfoliating process was employed to transfer the PMN-PT metal-insulator-metal (MIM) layer from the Si wafer without mechanical damage by an electroplated tensile Ni stressor. (iii) The PMN-PT film was placed on a PET plastic substrate using PU as an adhesive. UV light was then exposed to cure the PU between the PMN-PT film and PET substrate to achieve sufficient flexibility. (iv) Cu wires were attached to this flexible PMN-PT device to stimulate a live rat heart. The flexible PMN-PT film energy harvester was bent by tweezers without any damage as shown in Figure 8b and was attached to a glass tube with a radius of 1.4 cm (inset of Figure 8b). The phase of the PMN-PT film was analyzed by the Raman vibrational spectrum with a wavelength of 514 nm (Figure 8c). The Raman peaks observed at 120, 275, 578 and 785 cm^−1^ are associated with the F_2g_, F_2g_, E_g_ and A_1g_ modes, respectively, and are the typical signature for a perovskite relaxor of PMN-PT [110].

The rectified output current of the flexible PMN-PT harvester was found to be 100 µA, as shown in Figure 8d. This output current is high and sufficient to directly operate commercial electronics without any storage systems. The power is generated from the preset energy harvester by bending and unbending powered 50 green LEDs (inset of Figure 8d). In addition, a coin cell charged by continual bending of the device from 0 to 1.7 V in 3 h and an equivalent circuit connection comprising four diodes and a battery for energy storage are schematically represented in the inset of Figure 8e. The required driving voltage to operate an electronic device/stopwatch is 3 V. This type of energy harvester and storage system is promising as a new energy source in artificial pacemakers by increasing the battery size/replacing discharged batteries [111,112]. Moreover, the instantaneous electric output of the harvester was demonstrated to directly stimulate a rat heart without any external power circuits. The opened chest of a rat for stimulation of the heart and perception of a heartbeat is shown in Figure 8f. The corresponding spike peaks on the natural heartbeat of the rat were observed in the (electrocardiogram) ECG when the flexible PMN-PT stimulating device bent and unbent periodically, as shown in Figure 8g. The generated energy of 2.7 µJ from the stimulator by one bending motion is higher than the threshold energy of 1.1 µJ needed to stimulate the living heart [113]. These results of the PMN-PT energy harvester are promising for biomedical applications such as self-powered cardiac pacemakers [114].

#### 2.2.3. Transferring Single-Crystalline Piezoelectric Film onto a Plastic Substrate by Solid-State Single-Crystal Growth Method for a Flexible and High-Performance Energy Harvester

Many researchers have investigated flexible piezoelectric composite and film-based energy harvesters with novel methods and materials, as discussed in the above sections [25,26,37,46]. However, they have exhibited insufficient output power for driving mW-level electronics/high input current driven devices (~100 µA), limiting the range of applications [29]. To address this issue, a flexible energy harvester was realized by transferring a single crystalline piezoelectric PMN-PT film grown by the Bridgman method onto a PET substrate by a mechanical exfoliating process and provided enhanced output current of 145 µA by bending motion (Section 2.2.2) [27]. However, it has some drawbacks such as difficulty in controlling the growth orientation, cost-effective process of high-temperature melting and compositional inhomogeneity. In this regard, a research team reported a flexible and high-performance energy harvester fabricated by a single crystalline piezoelectric PMN–PZT film grown by the solid-state single crystal growth (SSCG) method [28].

**Figure 9:**
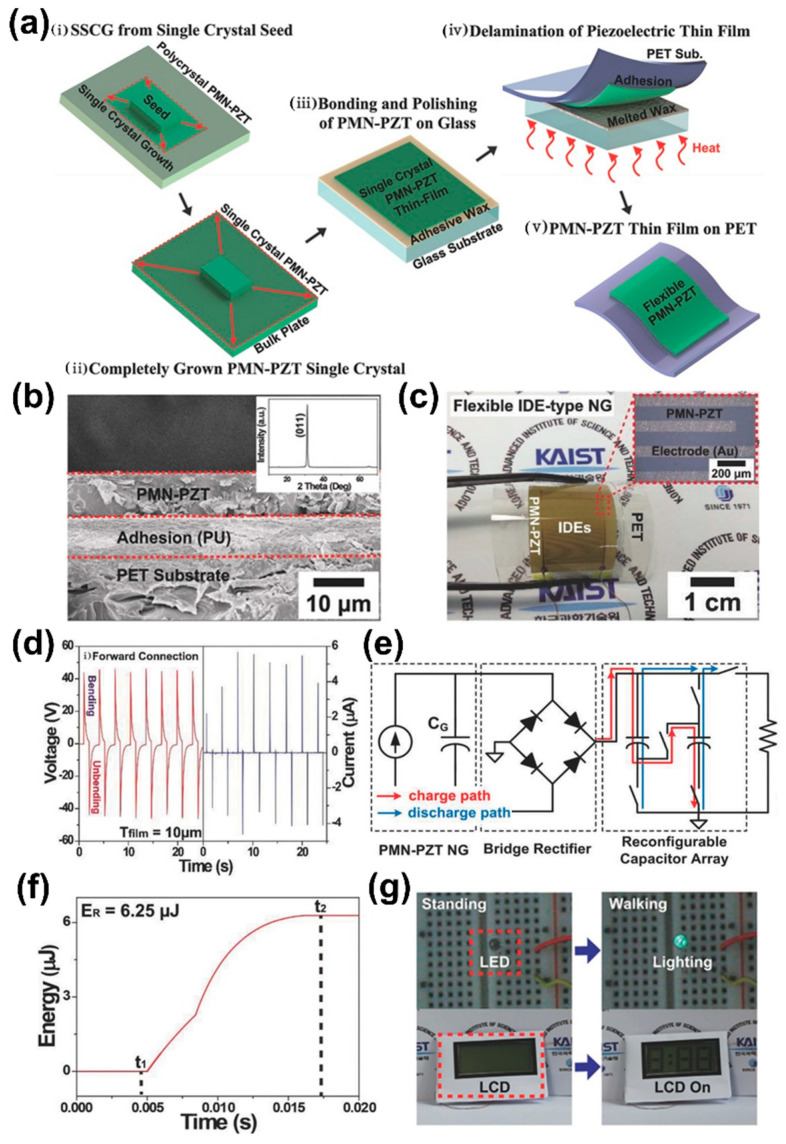
(**a**) Schematic diagram of the SSCG and fabrication method of a flexible PMN−PZT energy harvester. (**b**) Cross−sectional SEM image of PMN−PZT film on PET substrate. (**c**) Photograph of flexible PMN−PZT film energy harvesting device was bent by tweezers. (**d**) The generated voltage and current signals from the energy harvester during continual binding and unbending in forward connection. (**e**) Schematic circuit diagram of the designed reconfigurable rectifying circuit system composed of a flexible PMN−PZT energy harvester, bridge rectifier, external load resister and reconfigurable capacitor charger array. (**f**) The total converted heating energy from the reconfigurable rectifying system by one bending or unbending motion of the flexible PMN−PZT energy harvester. (**g**) The self−powered military boot powered a commercial LED and LCD with human walking. Reproduced with permission [28].

The SSCG and fabrication process of the flexible PMN–PZT energy harvester are explained as follows and schematically shown in Figure 9a. (i) A (011) oriented single crystalline PMN-PZT bulk block was grown by employing the SSCG method [107]. A (011) oriented small crystalline Ba(Zr_0.1_Ti_0.9_)O_3_ plate was placed on sintered polycrystalline PMN-PZT block as a seed template at 1200 °C for the process of SSCG. (ii) The small crystal seed grew into a large crystal by absorbing the polycrystalline body with grain growth. (iii) This single crystal PMN-PZT bulk was bonded onto a glass substrate using adhesive wax and thinned to 10 mm thickness by a polishing process. (iv) The PMN-PZT film on glass was placed on a PET substrate using PU as an adhesive epoxy and then UV light was exposed to cure the PU between the PMN-PZT film and PET substrate. The sample was heated at the melting temperature of adhesive wax (60 °C) to separate the area of the PMN-PZT film from the glass. The low temperature enabled a simple and robust technique to delaminate the piezoelectric thin film from the mother substrate without mechanical damage. (v) The PMN–PZT film was transferred onto the PET, IDEs were coated on the flexible PMN-PZT/PET film and finally Cu wires were connected to the electrodes to fabricate a flexible energy harvester.

A cross-sectional SEM image and the XRD pattern (inset) of the PMN-PZT film on a PET substrate are shown in Figure 9b. The SEM results revealed a uniform microstructure without any cracks, or delamination/waviness by introducing UV light and heat treatment during fabrication [18]. The XRD results show only a (011) oriented single crystalline structural characteristic. The flexible PMN-PZT film energy harvester was bent by tweezers without any damage, as shown in Figure 9c and a top-view optical image of the IDE pattern on the PMN–PZT film (inset). The generated V_oc_ and I_sc_ from the PMN-PZT device reached 45 V and 5.8 µA during periodical bending and unbending motions by the machine in a forward connection (Figure 9d). These results are outstanding compared to earlier reports of ZnO, BT and PZT-based flexible energy harvesters [22,25,26,33].

A reconfigurable rectifying circuit was developed to convert maximum electric energy from the harvester to minimize the trade-off problems such as energy conversion and supplying voltage. A schematic circuit diagram of the designed reconfigurable rectifying circuit system consisting of a flexible PMN-PZT harvester, bridge rectifier, external load resistor and reconfigurable capacitor charger array is shown in Figure 9e. The reconfigurable capacitor array and the energy harvester are impedance matched during the charging stage to maximize energy extraction while minimizing energy loss. During the discharging stage, the capacitors are rearranged to supply the required constant low-voltage into the external load. Further, it was compared with a conventional rectifying circuit to evaluate the energy conversion performance. These results showed that the reconfigurable rectifying circuit, which has a Joule heating energy of 6.25 µJ as shown in Figure 9f, is able to transmit four times higher electric energy to the load resistor than the conventional circuit (1.54 µJ). This shows a notable enhancement in the efficiency of energy conversion and can be utilized in AC-pulsed piezoelectric harvesters for commercial electronics. In addition, a flexible PMN-PZT energy harvester was demonstrated as a self-powered military boot that can convert human movement into electricity for commercial electronics. The self-powered military boot directly enabled a LED and LCD by the motion of a human ankle without any external power/circuits (Figure 9g).

#### 2.2.4. Transferring an Aerosol Deposited Piezoelectric Film onto a Plastic Substrate by an Inorganic-Based Laser Lift-off Process for a 2Flexible and High-Performance Energy Harvester

The single crystalline piezoelectric PMN-PT and PMN-PZT films were demonstrated to realize flexible piezoelectric energy harvesters for self-powered electronics and generated instantaneous mW-level power to operate various electronic and biomedical devices [27,28]. However, these flexible piezoelectric energy harvesters could be difficult to commercialize due to the high cost of producing single crystals [18,115]. Recently, there have been attempts to fabricate cost-effective energy harvesting devices in IoT applications and other functional devices with ZnO nanorods using extensive growth strategies, which have shown better piezoelectric performance [116,117,118]. In this regard, a cost-effective high-performance flexible piezoelectric energy harvester was demonstrated by a research group using an aerosol deposition (AD) based PZT film to develop a self-powered IoT system [119].

**Figure 10:**
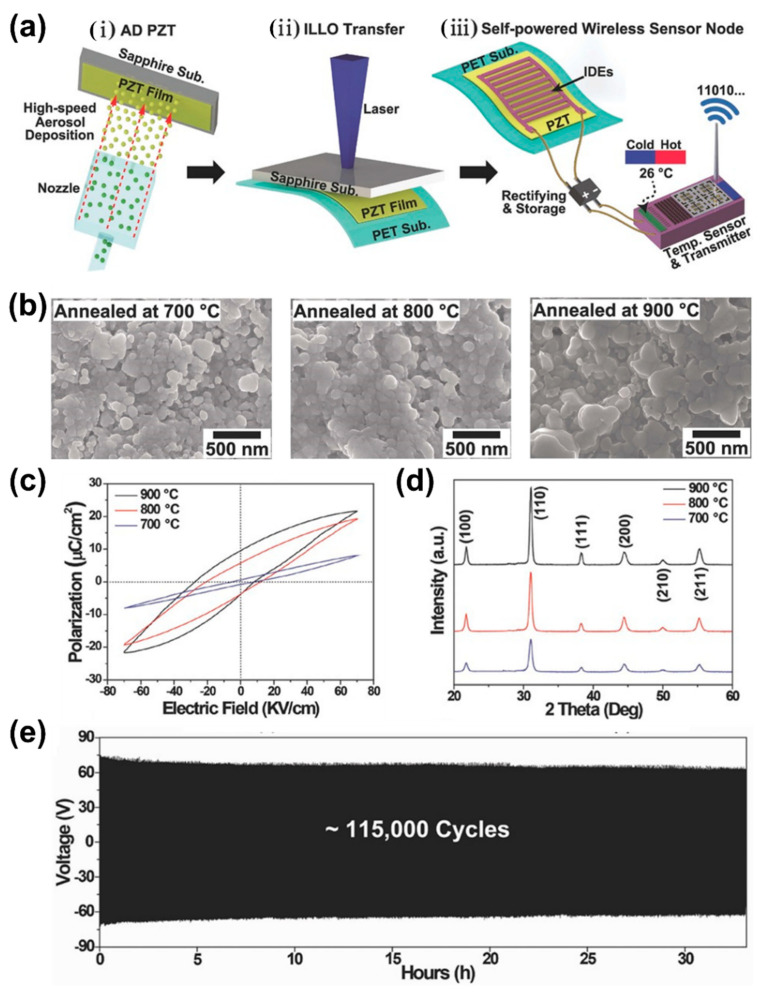
(**a**) Schematic diagram of the device fabrication and self−powered IoT using flexible AD PZT film by ILLO process. (**b**) SEM image of AD PZT films grown on sapphire wafers and annealed at 700, 800 and 900 °C for 1 h. (**c**) P−E hysteresis loops of annealed PZT films. (**d**) XRD patterns of annealed PZT films. (**e**) The bending durability test results of the flexible energy harvester for 115,000 cycles. Reproduced with permission [119].

The fabrication process and self-powered IoT by a flexible AD PZT energy harvester are explained as follows and schematically illustrated in Figure 10a. (i) The PZT film (7 µm) was deposited on a sapphire substrate by the AD process. The PZT particles interact with the carrier gas in the granule feeding chamber. The accelerated granules are then ejected under vacuum from the spray nozzle and bombard onto the sapphire substrate at high speed and room temperature. Thereafter, the deposited film was annealed at various temperatures from 700–900 °C for 1 h to enhance the ferroelectric and piezoelectric responses. (ii) The annealed PZT film on the sapphire substrate was bonded onto a PET substrate using a PU adhesive and cured by UV light. Laser wavelength of 308 nm was irradiated on the backside of the sapphire substrate for the removal of the sapphire substrate from the AD PZT film. This induced local vaporization in the interface between the sapphire and PZT layer, which results in the separation of the PZT film on the PET substrate from the mother sapphire substrate. This inorganic-based laser lift-off (ILLO) [110] enables simple, stable and high quality transfer of inorganic films onto plastic substrates. (iii) IDEs and a protective layer were coated on PZT film and finally the Cu wires were connected to the metal electrodes of the PZT harvester for self-powered IoT.

The output performance of the piezoelectric energy harvester can be effectively improved by increasing the grain size with annealing temperature [120]. The AD PZT films on sapphire substrates were annealed at various temperatures of 600, 800 and 900 °C for 1 h and the structural, microstructural and ferroelectric properties were investigated using SEM, XRD and P-E hysteresis loop characterizations, respectively. The SEM studies displayed uniform microstructure and the average grain size was found to have increased from 61.6–125.4 nm with annealing temperatures from 700–900 °C (Figure 10b). It is known that the critical grain size of a perovskite PZT material is less than 100 nm, but the AD PZT film that displayed grain size above this would have superior properties to the bulk ceramics due to diminished negative interference between ferroelectric domains and grain boundaries [121]. The XRD analysis of all annealed films revealed a well-crystallized single-phase perovskite structure (Figure 10d). The remnant and saturation polarization of the PZT films improved along with annealing temperature (Figure 10c). When the grain size of a PZT film increases above the critical size, the coupling between the ferroelectric domains and the grain boundaries decreases the repulsive force of the neighboring domains. As a result, remnant and saturated polarization are enhanced [122]. The high-domain wall mobility at larger grain size enhances the ferroelectric and piezoelectric properties of PZT, leading to high performance in the piezoelectric energy harvester [120]. A bending durability test was performed to verify the mechanical stability of the flexible energy harvester and it displayed stable output without significant variation for approximately 115,000 bending cycles at an induced strain of 0.3% on a plastic substrate.

**Figure 11:**
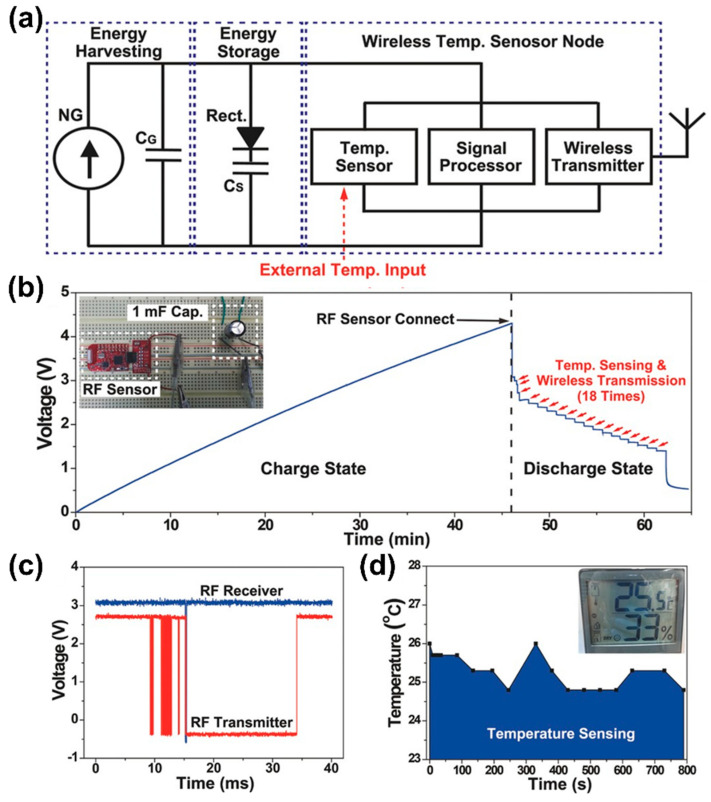
(**a**) Schematic circuit of self−powered temperature IoT system. (**b**) The charging and discharging response of a capacitor by PZT energy harvester for energy storage and operation of the temperature sensor node. (**c**) Output voltage measured from input/output pins of RF transmitter and receiver module. (**d**) The measured temperature was transmitted from the IoT system in 800 s. The digital thermometer shows the measured ambient temperature (inset). Reproduced with permission [119].

The self-powered IoT was fabricated and designed by integrating a flexible PZT energy harvester, bridge rectifier, storage capacitor and commercial IoT, as shown in Figure 11a. The generated AC current signal from the piezoelectric harvester under continual bending motions is converted to DC to charge a capacitor. The RF temperature sensor node receives power from the charged capacitor to measure ambient temperature. The information is transmitted wirelessly to a receiver connected to a computer and the measured data are displayed on a monitor. The generated power from the harvester was used to charge the capacitor from 0 to 1.3 V in 45 min and was discharged 18 times to operate the temperature wireless sensor node (Figure 11b). The RF sensor module and capacitor connections on a breadboard are shown in the inset of Figure 11b. In order to verify the operation of wireless communication, the output voltage was measured from external digital input/output pins available on both transmitter and receiver target boards. The output voltage of the input/output pin on each target board is depicted in Figure 11c. In the transmitter, the voltage dropped from 2.7 V to −0.4 V and remained low for 20 ms, which confirms the complete operation of temperature sensing, signal controlling/modulation, analog-to-digital conversion, bandpass filtering and RF signal transmission. In the receiver, the voltage dropped from 3 V to −0.5 V. The drops in the simultaneous voltage demonstrate that both transceivers mutually transmit and receive the RF signal, confirming that the two boards are capable of wireless communication. Figure 11d shows the variation of room temperature as a function of time in 800 s of telecommunication from the RF temperature sensor and ambient temperature measured using a digital thermometer to confirm the accuracy of sensing (inset). These findings confirmed the viability of a flexible AD PZT piezoelectric energy harvester to operate a self-powered IoT device for human healthcare or personal electronic devices.

#### 2.2.5. Self-Powered Wireless Transmission Enabled by Harvesting In Vivo Biomechanical Energy with Flexible and High Performance of a Single-Crystalline Piezoelectric Energy Harvester

The implantable biomedical devices used in ubiquitous healthcare systems can sense vital signs and transmit data [123,124]. In contrast, wireless data transmission significantly increases power consumption, thereby reducing battery lifetime [125]. Thus, additional surgeries are inevitable to replace discharged batteries of implantable devices and repeated surgeries can endanger patients by causing bleeding, inflammation and infection [126]. Therefore, many researchers have developed various power supplies for implantable medical devices such as wireless charging and photovoltaic power, which have some drawbacks such as electromagnetic damage and low energy efficiency in the human body [127,128,129]. Converting biomechanical energy into electrical energy to power implantable biomedical devices is a reasonable solution, since internal organs such as the heart, lungs and diaphragms have numerous mechanical movements [130,131]. Recently, flexible highly efficient piezoelectric energy harvesters have been demonstrated using single crystals for applications in implantable biomedical devices [16,28,67,105,132]. However, these reports investigated the operation of medical devices using in vitro energy harvesting from the bending stage. In this regard, Kim et al. demonstrated self-powered wireless data transmission operated by harvesting in vivo biomechanical energy with a high-performance piezoelectric single-crystalline PMN-PZT energy harvester in a big animal model [133].

**Figure 12:**
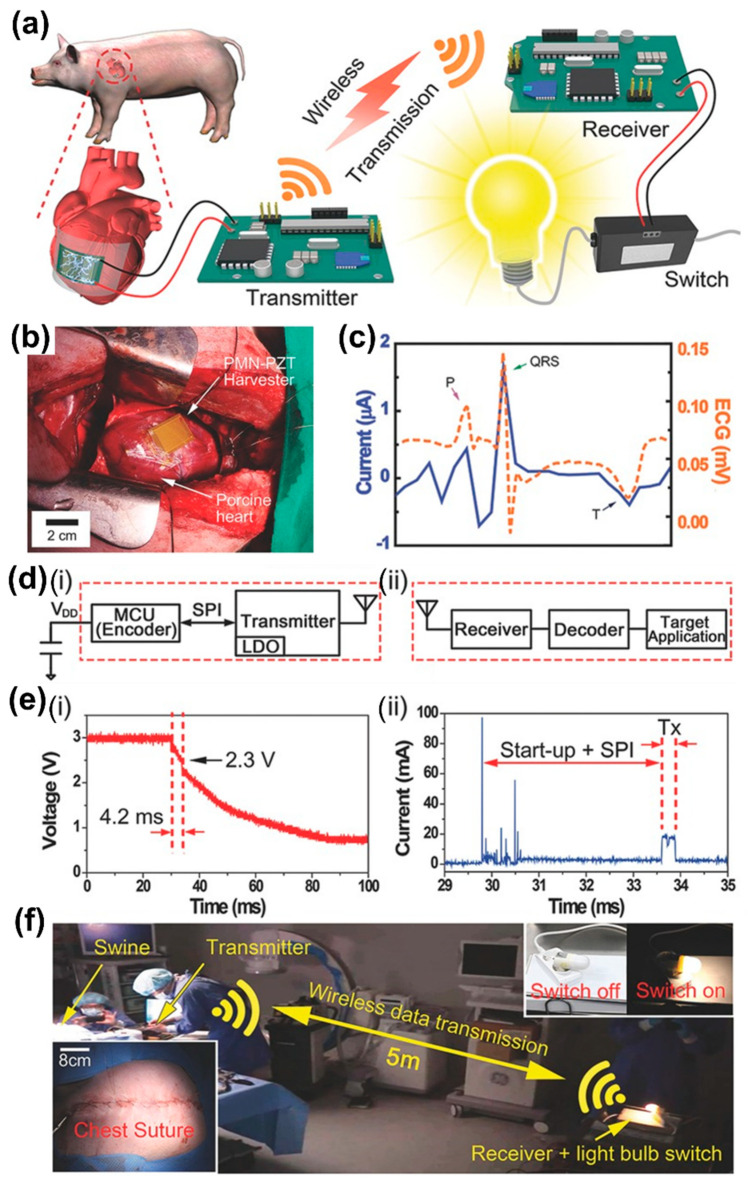
(**a**) Experimental schematic representation of in vivo self−powered wireless data transmission using flexible a PMN−PZT-Mn energy harvester attached to a porcine heart. (**b**) Photograph of flexible PMN−PZT−Mn harvester attached to a porcine heart. (**c**) Magnified current and ECG response from the porcine heartbeat. (**d**) Circuit diagrams of the wireless communication module consisting of (**i**) transmitter and (**ii**) receiver parts. (**e**) Specifications of the wireless communication module. (**f**) Photograph of big animal experiment. Reproduced with permission [133].

The PMN-PZT with Mn (0.5 mol%) doped (PMN-PZT-Mn) single crystal displayed a high piezoelectric charge coefficient and high electromechanical factor compared with that of other PZT materials [134,135]. The figure of merit (FOM) is an important factor and was developed to emphasize the selection of piezoelectric materials for energy harvesting applications [136,137]. The FOM of PMN-PZT-Mn was found to be higher than that of pure PMN-2PZT due to its high mechanical quality factor [135]. Therefore, the flexible PMN-PZT-Mn energy harvester was fabricated by the SSCG method and the fabrication is similar to that in a previous report (Section 2.2.4). An experimental schematic representation of in vivo self-powered wireless data transmission using a flexible PMN-PZT-Mn energy harvester attached to a porcine heart is shown in Figure 12a. An adult pig cardiac structure similar to that of humans was used as a big animal model to simulate the viability of energy harvesting in a human body [138]. The energy produced by the continuous heartbeats of a pig was stored in a capacitor by a full-wave bridge rectifier and utilized to perform wireless data transfer. The written data are transmitted wirelessly to the receiver using a wireless universal serial bus. The data were written as instructions for turning on and off a light bulb at a distance of 5 m in order to visually check the wireless data transmission. The implanted flexible PMN-PZT-Mn energy harvester in a porcine heart (adult male pig and weight of 40 kg) is shown in Figure 12b. The generated output current signals and ECG of the flexible PMN-PZT-MN energy harvester from the porcine heart are presented in Figure 12c. The generated V_oc_ of 17.8 V and I_sc_ of 1.75 µA are a factor of 4.45 and 17.5 higher, respectively, than earlier reported values in vivo piezoelectric energy harvesting [24]. The enhanced output current of this device is attributed to the piezoelectric charge coefficient and minimal thickness dependence of third-generation single crystals and is promising for practical application in wireless communication, sensing and pacing.

The circuit diagram of the wireless communication module consisting of transmitter and receiver parts is shown in Figure 12d. The voltage of the capacitor charged from the energy harvester is utilized to drain the voltage (V_DD_) of a transmitter (Figure 12(d-i)). After receiving the drain voltage, the microcontroller unit (MCU) started encoding communication data, sent to the transmitter from MCU and the data are transmitted wirelessly to the receiver as RF signals via an antenna. The receiver transformed the RF signals into digital signals when the receiver received the data (Figure 12(d-ii)). The data were delivered to a target application that was utilized to turn on and off a light bulb after being decoded by a decoder. The steep temporal slope from 29 to 34 ms of the voltage change of V_DD_ caused by wireless transmission reveals that the data were sent wirelessly as shown in Figure 12(e-i). The communication module needed a threshold voltage above 2.3 V to operate and hence the voltage decrease after 34 ms was caused by an input voltage beyond its normal range. During wireless communication, current was supplied to the module from 29 to 35 ms (Figure 12(e-ii)). The actual transmission took 0.4 ms from the start-up and serial peripheral interface (SPI) process, while the data transmission prework took 3.8 ms. It was concluded that a few ms are required to transmit the data and the PMN-PZT-Mn harvester is capable of generating the charge with in vivo energy harvesting. The big animal experiment used a median sternotomy on a pig to harvest energy in vivo, as shown in Figure 12f. The flexible energy harvester was attached to a porcine heart and worked well even after the chest closed (left inset of Figure 12f). A capacitor was connected in parallel to the device by a wire extending from the chest wall and the transmitter is connected to the capacitor again in parallel. When the electricity was charged in the capacitor above the threshold voltage, the light bulb at 5 m distance from the transmitter was wirelessly turned on and off without the use of any other power sources (right inset of Figure 12f). These results confirm that wireless data transmission was successfully operated by biomechanical energy.

#### 2.2.6. Transferring Aerosol Deposited Relaxor Ferroelectric and Piezoelectric Thick Films onto a Plastic Substrate by an Inorganic-Based Laser Lift-Off Process for a Flexible Self-Charging, Ultrafast and High-Power Density Ceramic Capacitor

Many researchers have investigated flexible energy conversion and storage devices, that can convert mechanical energy into electrical energy by piezoelectric/triboelectric energy harvesters and store that energy in a cell [139,140,141,142,143,144,145]. Self-charging power systems were developed by integrating energy harvesting and storage devices to operate self-powered IoT, wearable and biomedical electronic devices [146,147,148,149,150,151,152,153]. Many mechanisms have been investigated to develop self-powered systems, as discussed in the above sections. However, they have a few drawbacks such as low output power density, energy conversion efficiency, slow charging/discharging process and bulky complex designs, which prevent future pulsed-power systems of IoT, as well as wearable and implantable applications [154]. In this regard, Mahesh et al. [155] reported a novel approach to fabricate a flexible self-charging, ultrafast and high-powered density (SUHP) capacitor system by integrating an AD RFE PMN-PT capacitor and a piezoelectric PZT harvester. The AD PMN-PT relaxor film was used as an energy storage capacitor because of its superior energy-charging/discharging capabilities, slim hysteresis loop and relaxor properties. The PZT film with high piezoelectric responses was used as an energy harvester to achieve excellent output performance for even tiny mechanical motions.

**Figure 13:**
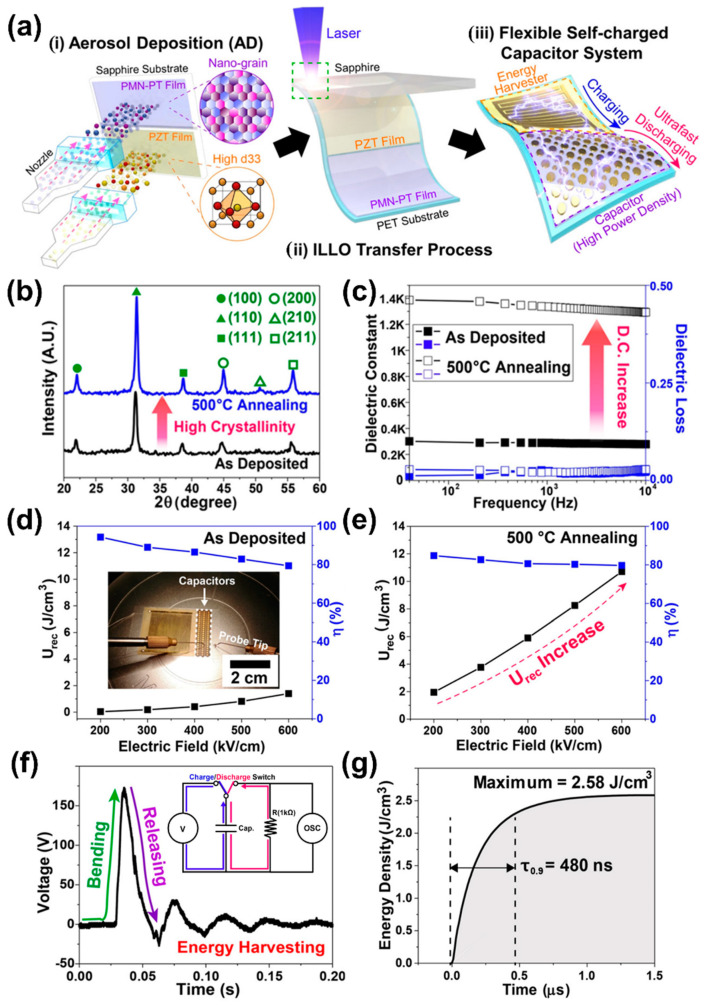
(**a**) Schematic representation of the fabrication process of flexible SUHP capacitor system. (**i**) Aerosol deposition of PMN−PZT and PZT films on sapphire substrates. (**ii**) Transferring PMN−PT and PZT films on sapphire substrates onto a flexible PET substrate by ILLO process. (**iii**) Deposition of IDEs, MIM electrodes and protective layer for flexible SUHP capacitor system. (**b**,**c**) XRD patterns and dielectric properties as a function of the frequency of as-deposited and annealed PMN−PT films. Recoverable energy density (U_rec_) and energy storage efficiency (η) response versus applied electric field of (**d**) as−deposited and (**e**) annealed PMN−PT thick film capacitors. (**f**,**g**) The output voltage response and discharged energy density from the SUHP capacitor system under bending/unbending motions by human fingers. A circuit diagram of the charging−discharging measurement system is shown in the inset of (**f**). Reproduced with permission [155].

Fabrication of the SUHP capacitor system is schematically shown in Figure 13a and was explained in three steps: (i) 0.9 PMN-0.1 PT and PZT films were deposited separately on sapphire substrates using the AD process and annealed at optimized temperatures of 500 and 900 °C for 2 h, respectively to improve their ferroelectric and piezoelectric responses by grain growth and crystallization. (ii) Both PMN-PT and PZT crystalline films were transferred from sapphire substrates onto a single PET substrate by the ILLO process. (iii) IDEs, MIM electrodes and a protective layer were grown on flexible PMN-PT and PZT films to fabricate the SUHP capacitor system on a PET substrate. Further, the fabricated energy harvester was poled by applying a field of 7 kV/mm at 70 °C for 3 h to enhance the piezoelectric responses of PZT. The XRD patterns of as-deposited and annealed PMN-PT films are shown in Figure 13b, which revealed the crystallized phase with a pure perovskite of PMN-PT. The dielectric constant (ε_r_) and dielectric loss (tanδ) as a function of the frequency of flexible PMN-PT films measured in a range of 40 to 10 kHz at room temperature, as shown in Figure 13c. The annealed film showed a higher ε_r_ (1335 @ 1 kHz) and a low tanδ (2.3% @ 1 kHz) than the as-deposited film (288 and 2.1% at 1 kHz). This was attributed to enhancement of the crystallinity and larger crystallite size, which can favor motion of the domain wall in the ferroelectric material.

The energy storage properties such as recoverable energy density (U_rec_) and energy storage efficiency (η) of as-deposited and annealed PMN-PT thick film capacitors were calculated from P-E hysteresis loops [156,157]. U_rec_ and η as a function of the applied electric field of both as-deposited and annealed films are shown in Figure 13d,e, respectively. A photograph of the P-E hysteresis loop measurement of a flexible PMN-PT film capacitor is depicted in the inset of Figure 13d. The U_rec_ value of as-deposited and annealed films was found to be 1.4 and 10.7 J/cm^3^, respectively, at an electric field of 600 kV/cm. The U_rec_ value was improved by nearly 7.6 times with the annealing process as compared to the as-deposited, which is a much higher value than earlier reports on a flexible polymer-based capacitor at 600 kV/cm [158,159]. In addition, the annealed film capacitor displayed a stable η (~79%) as a function of an electric field, whereas the as-deposited film revealed a comparatively high drop of η with an increasing electric field. The improvement of the energy storage properties of annealed samples is attributed to the dense microstructure with more nanograins caused by a large number of grain boundaries with lower conduction loss at high electric fields [160].

To verify the self-charging performance of a flexible SUHP capacitor system, the output performance of a flexible PZT energy harvester was demonstrated under mechanical bending/unbending motions using human fingers. The output voltage response from the PZT harvester as shown in Figure 13f, generated maximum V_OC_ and I_SC_ of 172 V and 21.6 µA, respectively. The working principle of the self-charging capacitor system is schematically illustrated in Figure 13a-iii, where the system is composed of a PZT harvester that acts as a power source and a PMN-PT capacitor as an energy storage device. The external microswitch allows for energy storage or discharge mode with a connecting load resistance of 1 kΩ, (inset of Figure 13f). The generated electrical voltage (172 V) from the PZT harvester by continual bending and unbending motions was quickly transferred to the PMN-PT capacitor through a micro switch. After charging the capacitor with harvested energy, the stored energy in the PMN-PT capacitor was discharged via a load resistor. The discharging response of the flexible SUHP capacitor as a function of time is shown in Figure 13g, it instantly discharged the storage energy with a recoverable energy density of 2.58 J in a discharge time of 480 ns. The outstanding performance of the flexible SUHP capacitor indicates that this new approach provides a platform for sustainable pulsed-power sources for operating flexible electronic devices [161,162].

## 3. Conclusions

**Figure 14:**
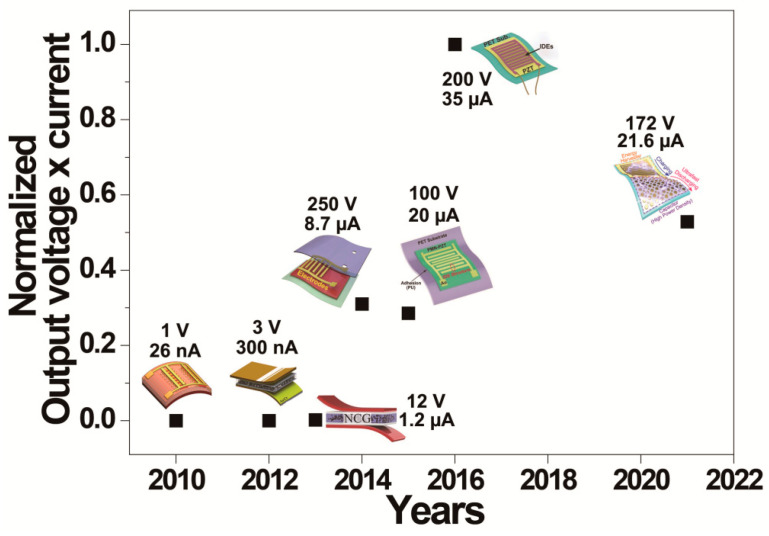
A summary on the output performance values of perovskite piezoelectric-based flexible energy harvesters over the last decade. The normalized output voltage and current values are calculated by multiplication of measured output open-circuit voltage and short-circuit current from bending motion of flexible piezoelectric energy harvesters. Reproduced with permissions.

This review has focused on flexible piezoelectric film and composite energy harvesters that have been developed for self-powered implantable and wearable IoT applications. The flexible piezoelectric films and composites on plastic substrates can provide energy conversion of tiny mechanical movement of the human body into an electric signal, thus enabling utilization as high-performance flexible energy source for various implantable and wearable IoT applications. First, low-cost and simple processes including spin-coating, die-casting and bar-coating allow the realization of flexible large-area piezoelectric composite generators. Typical inorganic-based perovskite piezoelectric particles such as BT, KNLN, PMN-PT, BCZT, PZT, PMN-PT and PMN-PZT were utilized to fabricate the piezoelectric composite harvesters. Their application field has been flexible energy harvesters for self-powered IoT devices as well as flexible mechanical sensors for biomedical applications. Furthermore, an ultra-stretchable wearable composite generator was developed with a hyper-stretchable electrode layer of an Ag NW network to achieve close contact with the curvy surfaces of the human body. The piezoelectric flexible composite device can generate electric energy from biomechanical movements, sufficient to operate commercial electronics. Second, flexible piezoelectric film harvesters with highly piezoelectric coefficients of ceramic PZT and single crystal PMN-based materials have derived a noticeable enhancement of electric output for flexible piezoelectric harvesters. The electricity generated from the bending motion of flexible piezoelectric film devices was enough to not only operate an IoT sensor but also stimulate living heart muscle. Furthermore, the flexible piezoelectric harvester can scavenge meaningful electric power from the cardiac contraction and relaxation motions, which facilitated self-powered wireless data transmission. These technical developments would extend the application of flexible piezoelectric energy harvesters as permanent power source in implantable and wearable IoT devices to support or substitute conventional capacity limited batteries. Figure 14 shows a summary of the output of flexible perovskite piezoelectric energy harvesters over the last decade. The normalized values at the graph are calculated by multiplication of output open-circuit voltage and short-circuit current from bending motion of the harvesting device, which are utilized to compare the performance improvement of flexible piezoelectric harvesters. Since 2010, the output performance of flexible piezoelectric harvesters has been gradually increased by the adoption of novel perovskite piezoelectric materials, new device structures and the latest fabrication techniques, thus enabling them to become a strong candidate as a continuous power source of implantable and wearable IoTs.

## Data Availability

Not applicable.

## Abbreviations

IoT	Internet of things
WSNs	Wireless sensor networks
LED	Light emitting diode
BT	BaTiO_3_
PZT	Pb(Zr,Ti)O_3_
PMN-PT	Pb(Mg_1/3_Nb_2/3_)O_3_–PbTiO_3_
PMN-PZT	Pb(Mg_1/3_Nb_2/3_)O_3_–Pb(Zr,Ti)O_3_
NCG	Nanocomposite generator
NPs	Nanoparticles
SW	Single wall
MW-CNTs	Multi-walled carbon nanotube
RGO	Reduced graphene oxide
PDMS	Polydimethylsiloxane
p-NC	Piezoelectric nanocomposite
SEM	Scanning electron microscope
Voc	Open circuit voltage
Isc	Short circuit current
C_N_	Capacitance
R_L_	Load resistance
R_N_	Internal resistance
mWs	Milliwatts
KNN	(K,Na)NbO_3_
KNLN	0.942(K_0.480_Na_0.535_)NbO_3_-0.058LiNbO_3_
Cu NRs	Copper nanorods
ITO	Indium tin oxide
PET	Polyethylene terephthalate
HRTEM	High-resolution transmission electron microscopy
FFT	Fast Fourier Transform
LCD	Liquid Crystal Display
SEG	Stretchable elastic-composite generator
VAgNWs	Very long Ag nanowires
PEC	Piezoelectric elastic composite
VLNP	Very long nanowire percolation
SMG	Successive multistep growth
MEMS	Microelectromechanical systems
RFEs	Relaxor ferroelectrics
NDs	Nanodomains
PNRs	Polar nanoregions
XRD	X-ray diffraction
PVDF	Polyvinylidene fluoride
LLO	Laser lift-off
IDEs	Interdigitated electrodes
MPB	Morphotropic phase boundary
MIM	Metal-insulator-metal
PU	Polyurethane
ECG	Electrocardiogram
SSCG	Solid-state single crystal growth
AD	Aerosol deposition
ILLO	Inorganic-based laser lift-off
AC	Alternating current
DC	Direct current
RF	Radio frequency
FOM	Figure of merit
VDD	Drain voltage
MCU	Microcontroller unit
SPI	Serial peripheral interface
SUHP	Self-charging, ultrafast and high-powered density
Urec	Recoverable energy density
